# Supplementary material for: Nonadherence to anti-tuberculosis treatment, reasons and associated factors among pulmonary tuberculosis patients in the communities in Indonesia
Source: PLoS One. 2023 Aug 8;18(8):e0287628. doi: 10.1371/journal.pone.0287628 (PMC10409295; doi:10.1371/journal.pone.0287628)
Supplement: S2 File — (PDF) [file pone.0287628.s002.pdf]

INDONESIA GOVERNMENT
MINISRY OF HEALTH
NATIONAL INSTITUTE OF HEALTH RESEARCH AND DEVELOPMENT

SPTB12

TUBERCULOSIS PREVALENCE SURVEY 2013-2014

CONFIDENTIAL

barcode

**I. PARTICIPANT INFORNATION**					***Q 1 Filled by Receptionist, the others by Interviewer***
1	Name participant				
2	Age	☐☐ Year	3	Sex 1. Male 2. Female	☐
4	Address	*(completed until sub-district)*			
5	Number.Telephon / mobile phone		6	Date of data collection: (dd-mm-yy)	☐☐-☐☐-☐☐☐☐

**II. INDIVIDU HISTORY INFORMATION**					***Filled in by Interviewer***
**7**	What you highest education graduated ?	1. Never school<br>2. Not graduated/graduated elementary<br>3. Graduated elementary school	4. Graduate yunior high school<br>5. Graduated high school<br>6. Graduated D1/D2/D3	7. Graduated bachelor<br>8. Graduated master/doctor	☐
**8**	Have ever been diagnosed Diabetic Mellitus by medical doctor?			1.Yes 2.NO	☐
**9**	Are you currently smoking cigarette?			1.Yes 2.NO	☐
**10**	Have you ever been been living with TB patient in the last two years?			1.Yes 2.NO	☐
**11**	Have you ever been diagnosed Lung TB by a health professional?			1.Yes 2 .NO → **Q. 19**	☐
**12**	When were you diagnosed TB by a health professional in the last time? **IF DO NOT REMEMBER THE MONTH, FILL"88"**	Month ☐☐		Year ☐☐☐☐	
**13**	a. Were any sputum examinations done by health professional in the last time?			1.Yes 2.NO →**Q. 14**	☐
	b. If yes, what was the result?<br>1. TB 2. Non TB 3. Waiting result 8. DK				☐
**14**	a. Was there chest X-ray examination for TB?			1.Yes 2.NO →**Q. 15**	☐
	b. If yes, what was the result?<br>2. TB 2. Non TB 3. Waiting result 8. DK				☐
**15**	Have you been treated for TB?			1.Yes 2.NO →**Q. 18**	☐
**16**	Where did you first recieve TB treatment?<br>1. Public hospital<br>2. Private hospital<br>3. Health center<br>4. Private professiona	5. Private clinic<br>6. Paramedic practice (nurse,midwive)<br>7. Farmasy /medicine store / Warung<br>8. Other, specify..........................			☐
**17**	a. Are you currently still taking TB medicine?			1.Yes → **Q.19** 2.NO	☐
	b. If No, how long did you have you take the medicine? …………(months)				☐☐
**18**	What was your main reason did not take or stop medicine?				
	1. Declare cured by Health professional<br>2. Feel better/Not symptoms<br>3. Feel no better<br>4. Have no money<br>5. No transportation	6. No body can pick up the medicine<br>7. No medicine in the healt facility<br>8. Side effect the medicine<br>9. Make urine red<br>10. Others......................................			

Barcode

**III. SCREENING ELIGIBLE FOR SPUTUM COLLECTION BY SYMPTOMS**		***Filled in by Interviewer***	
**19**	Are you currently coughing ?	1. Yes 2. NO → Q.24	☐
**20**	If yes, How long have you been coughing ?	........................days	☐☐☐
**21**	Do you currently cough up blood/haemoptysis/sputum with blood?	1. Yes 2. NO	☐
**22**	a. Have you ever seeking treatment for the coughing?	1. Yes 2. NO →Q. 23	☐
	b. If yes, where did you go for the first treatment?<br>1. Public hospital<br>2. Private hospital<br>3. Health center<br>4. Private professional	5. Private clinic<br>6. Paramedic practice (nurse,midwive)<br>7. Farmasy /medicine store / Warung<br>8. Other, Specify...............................	☐
**23**	Do you currently cough up with sputum or phlegm?	1. Yes 2. NO	☐
**24**	Do you currently have fever?	1. Yes 2. NO	☐
**25**	Do you currently have chest pain?	1. Yes 2. NO	☐
**26**	Do you currently have drenching night sweats?	1. Yes 2. NO	☐
**27**	Do you currently loss apptite?	1. Yes 2. NO	☐
**28**	In the past month, have you unexpected lost weight?	1. Yes 2. NO	☐
**29**	Do you currently have shortness in breathing/ dyspNoe?	1. Yes 2. NO	☐
**30**	For women (age 15-49 year), are you currently pregnant ?<br>IF MAN CODE "0"	1. Yes 2. NO	☐
**31**	a. Are you willing to take thorax photo?	1. Yes 2. NO	☐
	b. If No, what is the reason?	....	
	Name of interviewer	Sign of interviewer	....
**IV. SCREENING ELIGIBLE FOR SPUTUM COLLECTION BY THORAX PHOTO**		***Filled by radiolog***	
**32**	Result thorax photo<br>See Q. 31 a	1. Abnormal in the parenchim & pleura<br>2. Normal in the parenchim lung & pleura<br>3.Other, specify..<br>4. No taking thorax photo	☐
	Name of thorax photo reader	Sign thorax photo reader	....
**V. CONCLUSION**		***Filled by team leader***	
**33**	Are sputum speciment collected?<br>***TAKING SPUTUM IF Q.20≥14 DAYS OR Q.21 CODE "1" OR Q.32 CODE "1"***	1. Yes 2. NO →Q 35	☐
**34**	Did the particpant submit sputum?	1. Yes, submit sputum<br>2. Refuse<br>3. Can not produce sputum	☐
**35**	Is there any illnes needed to be refered to near health facility?	1. Yes 2. NO	☐
**36**	Is the Participant eligible interviewed for KAP?	1. Yes 2. NO → **STOP**	☐

Barcode

**VI. KNOWLEDGE, ATTITUDE & PRACTICE (KAP)**		***Filled in by Interviewer***
**37**	Do you know how a person get TB?<br>**1 "YES SPONTANEOUS " ANYTHING ELSE? OTHER ANSWERS WILL BE READED (PROBING) 2 "YES" 3 "NO" 8 "DK"**<br>a. Being hit on the chest ☐ f. Air polution ☐<br>b. Black magic/ curse ☐ g. Eating and drinking with TB patient ☐<br>c. Hereditary ☐ h. Using the same utensil together with TB patient ☐<br>d. Food / drink ☐ i. Through public servie equipment (WC, public telephone micropone etc] ☐<br>e. Exposure to sputum cough of TB patient ☐ j. Shakehand ☐<br>k. Other, specify........	
**38**	What are the sign and symptoms of TB**?**<br>**1 "YES SPONTANEOUS " ANYTHING ELSE? OTHER ANSWERS WILL BE READED (PROBING) 2 "YES" 3 "NO" 8 "DK"**<br>a. Coughing ≥ 2 weeks ☐ f. Loss of weight ☐<br>b. Coughing with sputum ☐ g. Fever ☐<br>c. Coughing with blood ☐ h. Night sweat ☐<br>d. Shortness in breathing/dyspnoe ☐ i. Loss of appetite ☐<br>e. Chest pain ☐ j Other, specify....... ☐	
**39**	a. Do you think TB is curable? — 1. Yes 2. NO 8. DK	☐
	b.If YES, what are going to do for treatment?<br>1. Going to health provider 2. Going to non health provider 3. Treatment myself	☐
**40**	Do you know TB treatment give freely? SHOW THE TB DRUG — 1. Yes 2. NO 8. DK	☐
**41**	If any of your family have TB, are you going to keep in secret? — 1. Yes 2. NO 8. DK	☐
**42**	Have you ever smoke? — 1. Yes<br>2. No ➔STOP	☐
**43**	Are you currently still smoking ?<br>1. Yes, everyday ➔Q.45 2. Yes, sometime ➔ Q.46 3. No	☐
**44**	If no, when did you stop smoke? →Q 47 Year..................	☐☐☐☐
**45**	On average how many cigarette/tobaco you smoke everyday? ....cigarettes →Q 47	☐☐
**46**	On average how many cigarette/tobaco you smoke everyweek? ....cigarettes	☐☐
**47**	How old were you when you first starting smoking /tobaco? Year..................	☐☐☐☐

Name of team leader:	....	Sign of team leader	....
Name of editor:	....	Sign of editor	....
