# Supplementary material for: Nonadherence to anti-tuberculosis treatment, reasons and associated factors among pulmonary tuberculosis patients in the communities in Indonesia
Source: PLoS One. 2023 Aug 8;18(8):e0287628. doi: 10.1371/journal.pone.0287628 (PMC10409295; doi:10.1371/journal.pone.0287628)
Supplement: Attachment — Submitted filename: REBUTTAL LETTER TO REVIEWER.docx [file pone.0287628.s003.docx]

**REBUTTAL LETTER TO REVIEWER**

| **NO** | **REVIEWER** | **AUTHOR RESPOND** |
| --- | --- | --- |
| 1 | According to the principal aim of the study, the authors should review the conclusions of the work. According to the title, reference is made to reasons and factors associated with nonadherence to tuberculosis treatment. However, the conclusions do not meet this expectation. | We appreciate your review and we have rewritten the conclusion in the revised manuscript. |
| 2 | In the introduction, reference is made to the DOTs program; however, the procedure for controlling the treatment is not explained. And I think it is important in a work on adherence to treatment. This is not explained in the methods section either. | We have revised and added a description of the DOTS program in the introduction section lines 67 to 71. However, because this was a cross sectional study, we did not control for DOTS compliance in this survey. |
| 3 | Please, could the authors clarify what is the sense of "stratified multi-stage clusters sampling"? What were the inclusion criteria for introducing the patients in the study? What was the significance of "selected cluster"? | We edited and added information regarding stratified multistage cluster sampling to the method section lines 121-132 (track change document), as well as to the inclusion criteria lines 133-137. |
| 4 | How the participants in the study were selected? Were they enrolled consecutively? | We include an explanation in lines 138-140. |
| 10 | What was the usual duration of treatments and the used drugs? | We have made changes to lines 146-149. The duration of therapy under the Indonesian national tuberculosis treatment program is six months. |
| 11 | What was the timing of treatment regarding to the interview and inclusion in the Study? | The duration of therapy under the Indonesian national tuberculosis treatment program is six months |
| 12 | What was the criterion for non-adherence? | Criteria for non adherence are people with history of TB or take a treatment for TB but the duration of treatment less than six months |
| 13 | It would be helpful to define the terms that will be evaluated during the Study. Please, add. | This survey is not doing evaluation of the treatment, as the aims of the study was to study the prevalence, but not evaluate “the why”. |
| 14 | Were confirmed cases from possible cases considered separately in the analysis? | We consider the possible cases as confirm case. So We did the analysis for all cases as confirmed cases. |
| 15 | Please, clarify the criteria for non-adherence. Likewise, establish clear criteria on adherence to treatment and non-compliance. Also, the criteria to establish the categories of cured, treatment completed, lost, treatment failure (regardless of adherence to treatment) | Individuals having a history of tuberculosis or who are currently receiving treatment for tuberculosis but whose treatment duration is shorter than six months meet the criteria for non-adherence.  The term "treatment completed" refers to the completion of a six-month- of treatment.  Treatment failure occurs when a patient receives treatment for less than six months.  When respondents are lost before to the study's conclusion, leaving incomplete data is term of “lost” participants |
| 16 | In data analysis part, the definition criteria should be moved to the previous part. | done |
| 17 | In results, the first part is about proportion of non-adherence. However, it could be better to explain the most important findings of the study or, in any case, the number of cases included. Moreover, the authors used the expression of proportion when they talk about total number. Please, review. | done |
| 18 | Any enrolled patient was excluded of the analysis? | See consort in line number 191. Perhaps the consort could clarify the answer for your question (referred to the consort attached |
| 19 | The second part of results is called “factors associated with-non-adherence to anti-TB therapy”. It seems that the objective and subjective data were not analyzed separately. | *We have no intention about objective and non objective subjects. All we did was accordingly to the sample criteria and implementing the inform consent wich is agreed before the interview is taken.* |
| 20 | It seems that, patients in current TB are included in the study. It is true? In this case, please, would you clarify the term: "Non TB cases vs "Yes TB cases | We have written in methods section. Current TB status is the confirm results of TB examination at time of the survey (categorized as TB cases and non TB cases). |
| 21 | Regarding the “Current smoking”, it was a condition during the TB treatment or at the moment of completing the interview? | current smoking defined as still smoking at the time of the survey (yes or no). |
| 22 | Please, explain the differences about “health centers”, Government hospitals”, “private hospitals”, Private practitioners”. | Health center (the closest public health facilities in the community), government hospitals (the second layer of the public health facilities that can be used as the patient referral), private hospital (other health facilities belongs to the private sector), meanwhile the private practitioners defined as all of the clinics or health services by medic or paramedic including self-medication. |
| 23 | Regarding the interview, was the interview performed by patients or personnel | This interview performed by the patients/participants. We did a training for the interviewer in order to find a qualified data. |
| 24 | In relation to interrupted treatment, on the first line, the authors, talk about “interruption of TB medications before six months”, please, clarify. | Interupted treatments means that participants have received tuberculosis therapy in accordance with Indonesia's tuberculosis treatment program. Non-adherence TB was participants who had TB history and experience of TB treatment for less than six months. adherence TB was participants who had TB history and experience of the TB treatment for more than six months |
| 25 | On the discussion part, start with the more significant findings. It seems there are three elements: the proportion of non-adherence, the associated general factors and the “reasons b l for discontinuing treatment. Please, clarify every “part”. In my opinion it would be clearer to avoid mixture of data. | Done. We have rewritten on discussion part |
| 26 | Please, listening the associated factors or reasons related to non-adherence in the beginning of every part would be easier to know the findings of the Study. | Thank you |
| 27 | Was the survey data checked (for example, when they answered that the professional told them they were cured)? | Yes, we did several check, starting from enumerator define the participants as the proper subject. Later, a doctor will re-check for the consistency and completeness of the answer of the subject. The data validity was done double entry in the field by enumerators. Furthermore, another double entry done, followed by data cleaning and editing before the data run for the analysis of the primary study results. |
| 28 | On the conclusions, please, consider the title of the paper. The Study and the conclusions should respond the principal aim and expectative created by the title of the paper. Nonadherence factors are mentioned and the actions to reduce the interruption of treatment were introduced for first time. Please, think to talk about it in the discussion part, for instance and in the introduction. | we have rewritten the conclusion in the revised manuscript. |
| 29 | “Ethics statement” should be moved before the results, due to, in my opinion, is related to Methods. | We add ethics statements in line number 168-171 |
| 30 | Please, review the bibliography and the number of authors per reference. | done |
