# Supplementary material for: Nonadherence to anti-tuberculosis treatment, reasons and associated factors among pulmonary tuberculosis patients in the communities in Indonesia
Source: PLoS One. 2023 Aug 8;18(8):e0287628. doi: 10.1371/journal.pone.0287628 (PMC10409295; doi:10.1371/journal.pone.0287628)
Supplement: Attachment — Submitted filename: NEW REBUTTAL LETTER TO REVIEWER (1)_20102022.docx [file pone.0287628.s004.docx]

**REBUTTAL LETTER TO REVIEWER 20102022**

| **NO** | **REVIEWER** | **AUTHOR RESPOND** |
| --- | --- | --- |
| 1 | Reviewer #1: Thank you for the explanations and corrections on the paper. I don't have much more questions regarding it. Only a few details: On the part "Study design and participants", the sentence "**written informed consent was obtained and it was signed to all participants" could be written without bold letters.** On the part "Ethics statement" the informed consent process is explained again. | We have revised without bold letters  “The ethical clearance for the study was provided by the Ethics Committee of the NIHRD, number KE.01.10/EC/651/2012. This is secondary data analysis so that ethical clearance is no more needed. Written inform consent is applied before the interview was conducted and it was signed to all participants. All of the enumerators were trained to avoid bias of information. All identity details of the survey participants were kept confidential” |
| 2 | Finally, on "conclusions", at the end of first paragraph the authors say: "Reasons for non-adherence TB treatment depend on subject response and self-confession respectively". According to the methods it could be considered as limitation of the study, and it is reported in this sense on the part "limitations". | We have revised and added a limitation study  “The design of this study is cross-sectional so that the temporal relationship of several independent variables with the dependent variable cannot be ascertained. Non-adherence was assessed from the TB treatment history instead of hospital-based data. Consequently, participants might be subjected to recall bias. **Reasons for non-adherence TB treatment only based on subject response and self-confession as one of limitation study**”. |
| 3 | On the other hand, on the part of “References”: #1, please, the link to the document should be added. #2, please, the link to the document should be added. #4, please, the link to the document should be added. Please, review the reference #5. The authors are: Dick, J.; Lombard, C. Please, the link of #7 should be added. Please, the link of #11 should be added. Please, the link of #23 should be added. Please, review if it is necessary to add new links on the reference part, as, for instance, on #40 | We edited and added link in reference |
