# Supplementary material for: Nonadherence to anti-tuberculosis treatment, reasons and associated factors among pulmonary tuberculosis patients in the communities in Indonesia
Source: PLoS One. 2023 Aug 8;18(8):e0287628. doi: 10.1371/journal.pone.0287628 (PMC10409295; doi:10.1371/journal.pone.0287628)
Supplement: Attachment — Submitted filename: NEW REBUTTAL LETTER TO REVIEWER (1)_28122022.docx [file pone.0287628.s005.docx]

**REBUTTAL LETTER TO REVIEWER 28122022**

| **NO** | **REVIEWER** | **AUTHOR RESPOND** |
| --- | --- | --- |
| 1 | Reviewer : Please review your reference list to ensure that it is complete and correct. If you have cited papers that have been retracted, please include the rationale for doing so in the manuscript text, or remove these references and replace them with relevant current references.  Any changes to the reference list should be mentioned in the rebuttal letter that accompanies your revised manuscript. If you need to cite a retracted article, indicate the article’s retracted status in the References list and also include a citation and full reference for the retraction notice. | We have revised and added a links of reference. Revised in reference 33 and link for reference 35, we also removed reference 39 in previous draft manuscript |
