# Supplementary material for: Nonadherence to anti-tuberculosis treatment, reasons and associated factors among pulmonary tuberculosis patients in the communities in Indonesia
Source: PLoS One. 2023 Aug 8;18(8):e0287628. doi: 10.1371/journal.pone.0287628 (PMC10409295; doi:10.1371/journal.pone.0287628)
Supplement: Attachment — Submitted filename: NEW REBUTTAL LETTER TO REVIEWER (1)_10042023.docx [file pone.0287628.s006.docx]

**REBUTTAL LETTER TO REVIEWER 04 2023**

| **NO** | **REVIEWER** | **AUTHOR RESPOND** |
| --- | --- | --- |
| 1 | Reviewer : Please review your reference list to ensure that it is complete and correct. If you have cited papers that have been retracted, please include the rationale for doing so in the manuscript text, or remove these references and replace them with relevant current references.  Any changes to the reference list should be mentioned in the rebuttal letter that accompanies your revised manuscript. If you need to cite a retracted article, indicate the article’s retracted status in the References list and also include a citation and full reference for the retraction notice. | We have revised and added a links of reference. Revised in reference 33 and link for reference 35, we also removed reference 39 in previous draft manuscript |
| 2 | In some parts there is repetitions and data that could be expressed avoiding redundancies. For instance, please, review the introduction section and avoid terms or repetitive concepts. This action, will improve the comprehension and the ease of reading. Similar aspects should be reviewed on the part “Study design and participants”. Age, studies carried out, are repeated later. | We have revised, and reduce some repetition sentences in introduction, an methodology including study design and participant |
| 3 | On the last part, the paragraph: "the total sample involved in the survey..." should be moved to the part of results. In fact, now it is started doing reference to this data. Please, review the part of “measurement of variable” and avoid the duplicity of data. I have a few questions regarding the number of included patients. Were all patients included in the study? Were no patients lost during the study? No patients were excluded? | We have revised the manuscript following the suggestion, “total samples that was involved “move to results section. The number participant has explained in method and results section |
| 4 | Consider removing some of the non-essential data in the text, where the data is already in the tables. It makes reading more difficult and does not provide more information. respect to work. Regarding the changes performed in the text due to the revision process, some verbs and sentences should be changed now | We have revised in results section and avoid some repetition data on table and text |
| 5 | Please, clarify the second conclusion of this part. | We have revised the second conclusion in the end of manuscript |
